# One-Pot Radiosynthesis of [^18^F]Anle138b—5-(3-Bromophenyl)-3-(6-[^18^F]fluorobenzo[*d*][1,3]dioxol-5-yl)-1*H*-pyrazole—A Potential PET Radiotracer Targeting α-Synuclein Aggregates

**DOI:** 10.3390/molecules28062732

**Published:** 2023-03-17

**Authors:** Viktoriya V. Orlovskaya, Olga S. Fedorova, Nikolai B. Viktorov, Daria D. Vaulina, Raisa N. Krasikova

**Affiliations:** 1 N.P. Bechtereva Institute of the Human Brain, Russian Academy of Science, 197376 St. Petersburg, Russia; 2 St. Petersburg State Technological Institute, Technical University, 190013 St. Petersburg, Russia

**Keywords:** α-synuclein, fluorine-18, radiofluorination, 6-[^18^F]fluoropiperonal, [^18^F]anle138b, one-pot synthesis

## Abstract

Availability of PET imaging radiotracers targeting α-synuclein aggregates is important for early diagnosis of Parkinson’s disease and related α-synucleinopathies, as well as for the development of new therapeutics. Derived from a pyrazole backbone, ^11^C-labelled derivatives of anle138b (3-(1,3-benzodioxol-5-yl)-5-(3-bromophenyl)-1*H*-pyrazole)—an inhibitor of α-synuclein and prion protein oligomerization—are currently in active development as the candidates for PET imaging α-syn aggregates. This work outlines the synthesis of a radiotracer based on the original structure of anle138b, labelled with fluorine-18 isotope, eminently suitable for PET imaging due to half-life and decay energy characteristics (97% β+ decay, 109.7 min half-life, and 635 keV positron energy). A three-step radiosynthesis was developed starting from 6-[^18^F]fluoropiperonal (6-[^18^F]FP) that was prepared using (piperonyl)(phenyl)iodonium bromide as a labelling precursor. The obtained 6-[^18^F]FP was used directly in the condensation reaction with tosylhydrazide followed by 1,3-cycloaddition of the intermediate with 3′-bromophenylacetylene eliminating any midway without any intermediate purifications. This one-pot approach allowed the complete synthesis of [^18^F]anle138b within 105 min with RCY of 15 ± 3% (*n* = 3) and *A*_m_ in the range of 32–78 GBq/µmol. The [^18^F]fluoride processing and synthesis were performed in a custom-built semi-automated module, but the method can be implemented in all the modern automated platforms. While there is definitely space for further optimization, the procedure developed is well suited for preclinical studies of this novel radiotracer in animal models and/or cell cultures.

## 1. Introduction

Positron emission tomography (PET) is a sensitive and versatile imaging modality, often used in conjunction with CT or MRI, offering unique opportunity for a dynamic 3D-visualization of in vivo processes with a mm-scale resolution. The method employs molecular probes labeled with short-lived positron-emitting radionuclides (PET-radiotracers) interacting with specific protein targets of interest. Apart from widespread diagnostic application in oncology, PET has become an essential tool for assessing pathological processes in a variety of neurodegenerative disorders [1]. Parkinson disease (PD) is the second most widespread neurodegenerative condition worldwide, the prevalence of which is increasing as population ages. PD is characterized by progressive degeneration of the dopaminergic system with loss of dopaminergic neurons in the substantia nigra, associated with clinical presentation of motor symptoms. For in vivo assessment of the central dopaminergic function, the 6-l-[^18^F]fluoro-3,4-dihydroxyphenyl-alanine has been seen as the “gold standard” since 1983 [2]. Following metabolic pathway of l-DOPA, the ^18^F-fluorinated analogue crosses the blood-brain barrier and is subsequently decarboxylated to 6-[^18^F]fluorodopamine, which is accumulated within vesicles of dopaminergic neurons [3]. Synaptic dopamine levels are regulated by dopamine reuptake via dopamine transporter (DAT), whereas reduced levels of DAT indirectly reflect degeneration of nigrostriatal neurons [4,5]. PET imaging of DAT with [^18^F]FE-PE2I [6,7] enables the detection of presynaptic dopamine deficiency and provides another biomarker for PD progression assessment.

Together with the loss of midbrain nigrostriatal dopaminergic neurons, the pathologic processes in idiopathic PD and related disorders, such as dementia with Lewy bodies (DLB) and multiple system atrophy (MSA), are characterized by accumulation of α-synuclein aggregates [8,9,10]. α-Synuclein (α-syn) is an intrinsically disordered protein that is seen as a major component in Lewy bodies (LBs), Lewy neurites, and glial cytoplasmic inclusion type aggregates [11,12,13]. It has been assumed that a nigrostriatal dopaminergic tract related symptoms are preceded by LBs formation [11,12]. Development of molecular probes that enable in vivo visualisation and quantification of α-syn aggregates may allow for an earlier and more accurate diagnosis of PD and related α-synucleinopathies, and aid in the development of new treatments.

Over the past few years, extensive efforts have been focused on finding α-syn-specific PET radioligands (for the recent rev., see [13,14,15,16]). In 2022, the first human study on α-syn imaging using [^18^F]ACI12589 developed by AC Immune was reported [17]. The radiotracer is said to have high affinity and selectivity for α-syn and shows uptake in the brain areas (such as the basal ganglia and cerebellar white matter) known to be affected by pathological processes in patients with MSA—a rare, atypical parkinsonian syndrome. However, it remains unclear whether [^18^F]ACI12589 is able to detect α-syn depositions in the subjects with more common α-synucleinopathies, such as idiopathic PD and LBD [17].

**Figure 1:**
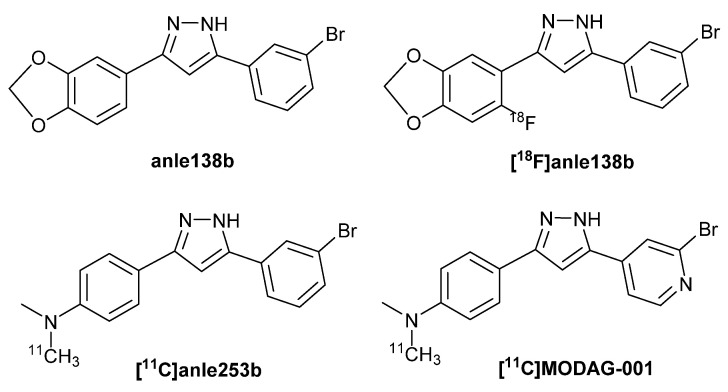
Chemical structures of 3,5-diarylpyrazole derivatives: anle138b, [^18^F]anle138b, [^11^C]anle253b and [^11^C]MODAG-001.

The importance of α-syn as a target continues to stimulate research in the area, and numerous studies are ongoing, with the aim to develop other labelled small molecules allowing for imaging of α-syn aggregates. Derived from pyrazole backbone, labelled analogues of anle138b (3-(1,3-benzodioxol-5-yl)-5-(3-bromophenyl)-1*H*-pyrazole) (Figure 1)—an inhibitor of α-syn and prion protein oligomerization [18,19]—are in active development as the candidates for α-syn imaging. Anle138b was designed for treatment of the rapidly progressing MSA and PD with potential to be applied to other synucleinopathies, such as DLB. It was demonstrated that anle138b interacts with aggregate forms of α-syn with moderate affinity (K_d_ of 190 ± 120 nM) [20] and strongly inhibited formation of pathological oligomers and neuronal degeneration in mouse models of α-syn and prion disease with improved survival rates [18,19,20,21]. The compound showed a high oral bioavailability and excellent blood-brain barrier penetration. In a range of different mouse models of PD and MSA, anle138b administration reduced protein aggregate deposition in the brain and improved dopamine neuron function and movement, even when treatment began after development of motor symptoms [21]. Recently, anle138b safety, tolerability and pharmacokinetics has been evaluated in healthy volunteers [22]. Phase I clinical trials in PD patients are currently ongoing.

The first PET tracer based on the lead structure from the library of the reported 3,5-diarylpyrazoles [18] has been the carbon-11 labelled anle253b—a molecule with a suitable position for ^11^C-labelling [23]. Evaluation of the radiotracer in healthy rats showed low brain uptake and suboptimal pharmacokinetics [23]. Soon after, the same group introduced a derivative of anle253b called MODAG-001 [24], in which one of the phenyl groups was replaced with pyridine. PET imaging in mice showed excellent brain uptake but detected the formation of two brain-penetrating radio-metabolites—something that hampers quantification of [^11^C]MODAG-001 uptake. To inhibit the metabolic demethylation process, a deuterated derivative (*d*3)-[^11^C]MODAG-001 was developed and was shown to be able to bind to pre-formed α-syn fibrils (α-PFF) in a protein deposition rat model [25]. However, no evidence of binding to aggregated α-syn was observed in human brain sections from DLB patients. Further evaluation of (*d*3)-[^11^C]MODAG-001 in a porcine brain with intracerebral injection of α-PFF and post-mortem human AD revealed that the radiotracer was not very selective for α-syn and exhibited significant binding in the AD regions [25].

Despite mixed results of anle138b/MODAG development when it came to carbon-11 labelling [24,25], we considered it worthwhile to attempt synthesis of a radiotracer based on the original structure of anle138b but labelled with longer-lived fluorine-18 isotope (T_1/2_ 110 min) which displays excellent decay characteristics for PET imaging (97% β+ decay, 635 keV positron energy). The structure–activity (SAR) studies revealed that the placement of bromine in *meta*-position of the 5-phenyl ring led to the highest inhibitory activity of anle138b, whereas further modification of that part of the molecule may result in reduced inhibition [18]. Therefore, in the present work, the 3-substituted aryl moiety was chosen as a suitable labelling position for [^18^F]anle138b (Figure 1). To introduce fluorine-18 into this non-activated position of the aromatic ring, a three-step radiolabelling strategy was attempted starting from 6-[^18^F]fluoropiperonal, obtained through different radiolabelling approaches. As suitability for automation is one of the most important requirements for the labelling method application, a one-pot procedure without intermediate purifications was developed and implemented in a semi-automated module. As a result, [^18^F]anle138b was obtained in 15.1 ± 2.3% (*n* = 3) radiochemical yield (decay-corrected) and *A*_m_ in the range of 31.5–79.5 GBq/µmol within a total synthesis time of ca. 105 min.

## 2. Results and Discussion

### 2.1. Radiolabeling Approach for [^18^F]Anle138b

The most common method for introduction of fluorine-18 into majority of PET radiotracers is an aliphatic nucleophilic substitution reaction between no-carrier-added [^18^F]fluoride and a precursor possessing a suitable leaving group in the presence of phase-transfer catalyst (PTC) to enhance [^18^F]fluoride reactivity. For aromatic compounds, the classical S_N_Ar method requires presence of a leaving group as well as an electron-withdrawing group, preferably in *ortho*- or *para*-position [26]—a requirement difficult to accommodate or engineer in case of complex compounds, such as anle138b. Therefore, the labelling often necessitates multi-steps “built-up” procedures where fluorine-18 is initially introduced into the aromatic ring of a simple and reactive substrate. All nucleophilic fluorinations begin with isolation of [^18^F]fluoride ion from proton-irradiated [^18^O]water; typically, this is achieved by trapping [^18^F]F^−^ on a quaternary ammonium ion-exchange resin followed by elution with a basic solution of a phase-transfer agent (e.g., Kryptofix K_2.2.2_/K_2_CO_3_ mixture in CH_3_CN/H_2_O). The [^18^F]fluoride ion complex with the PTC can then be dried to remove water and provide a reactive intermediate. In some instances, bulky counter-ions used for solubilisation of the [^18^F]fluoride, such as R_4_N^+^ (R = Et or *n*Bu), are introduced separately as salts (e.g., HCO_3_, tosylate, triflate); use of such phase-transfer catalysts can simplify trapping/elution procedures [27].

**Scheme 1:**
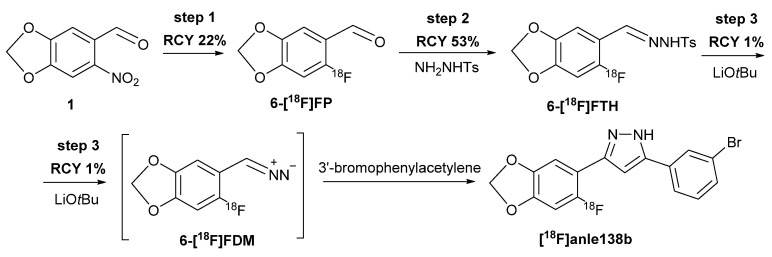
Proposed radiosynthesis route for [^18^F]anle138b (Zarrad, 2017) [29]; RCY—radiochemical yield, decay-corrected.

**Figure 2:**
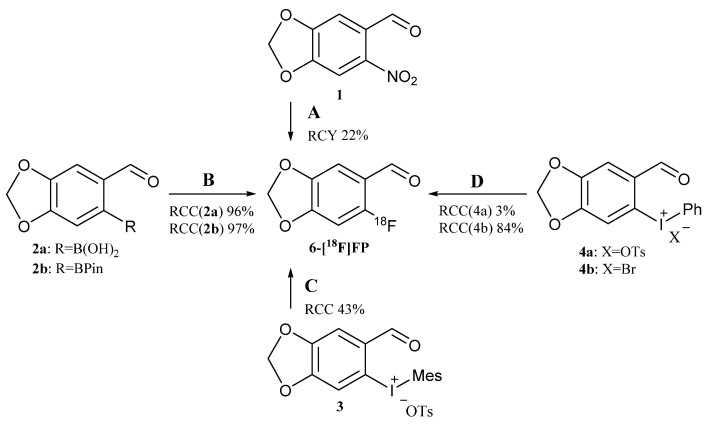
Radiolabelling routes to 6-[^18^F]FP. Conditions: (A) [29] (i) elution of [^18^F]F^−^ with Et_4_NHCO_3_ in MeOH; (ii) evaporation of MeOH; and (iii) **1** in DMSO, 130 °C for 10 min with two SPE and one HPLC purifications; (B) (i) elution of [^18^F]F^−^ with Bu_4_NOTf in 2-PrOH directly to the solution of **2a** or **2b** and Cu(OTf)_2_(py)_4_ in CH_3_CN; (ii) 65 °C for 10 min, and then 110 °C for 10 min; (C) (i) elution of [^18^F]F^−^ with Et_4_NHCO_3_ in MeOH; (ii) evaporation of MeOH; (iii) **3** and Cu(CH_3_CN)_4_OTf in DMF, 90 °C for 20 min; (D) (i) elution of [^18^F]F^−^ with **4a** or **4b** in MeOH/PC; (ii) 85 °C for 10 min, and then 120 °C for20 min; RCC—radiochemical conversion as determined by radioTLC; RCY—radiochemical yield corrected for radioactive decay; procedures (B) and (D) avoid any azeotropic drying or solvents evaporation steps.

The multi-step labelling strategy for [^18^F]anle138b—(5-(3-bromophenyl)-3-(6-[^18^F]fluorobenzo[*d*][1,3]dioxol-5-yl)-1*H*-pyrazole)—has been drafted by Zarrad et al. [28,29], and consisted of 1,3-cycloaddition between ^18^F-fluorinated phenyldiazomethane generated in situ from the corresponding tosylhydrazone with 3′-bromophenyl acetylene as a key synthesis step (Scheme 1). The ^18^F label was introduced into the commercially available precursor—4,5-methylendioxy-2-nitrobenzaldehyde (6-nitropiperonal, Step **1**, Scheme 1, Figure 2)— possessing carbonyl functional group in *ortho*-position, acting as an electron-withdrawing group. Fluorination was performed with Et_4_NHCO_3_ as a PTC and was followed by the isolation of the obtained 6-[^18^F]fluoropiperonal (6-[^18^F]FP) via semi-preparative HPLC and two solid-phase extraction (SPE) purifications. As a result, the 6-[^18^F]FP was obtained in 22% radiochemical yield (RCY, decay-corrected) with high radiochemical and chemical purity. Without this time-consuming and difficult to automate intermediate step aimed at the removal of unreacted 6-nitropiperonal, the condensation reaction of 6-[^18^F]FP with tosylhydrazide (Step 2, Scheme 1) would not proceed to yield [^18^F]anle138b. The suggested three step labelling procedure with intermediate purifications afforded [^18^F]anle138b with RCY of 1% (decay-corrected) which, while being sufficient for in vitro studies, would generally be seen as too low to be practical for most other uses [29].

Based on this proof-of-concept study, we have focused our efforts on the development of a simplified one-pot labelling procedure for [^18^F]anle138b with the aim of eliminating most, if not all, intermediate purification steps in the preparation of the 6-[^18^F]FP.

Taking into consideration the recent advances in radiofluorination of electron-rich aromatic strictures [30,31,32,33], we explored alternative routes to 6-[^18^F]FP using different radiolabelling precursors and conditions (Figure 2B–D).

### 2.2. Synthesis of 6-[^18^F]FP via Copper-Mediated ^18^F-Fluorodeboronation (Method B)

As a starting point, we investigated the feasibility of a copper-mediated approach using the commercially available catalyst Cu(py)_4_(OTf)_2_, originally developed for radiofluorination of pinacol arylboronates (ArylBPin) [34]. Its use was further extended to fluorination of organoborons [35] and (hetero)aryl organostannanes [36] and has been found to be useful in the preparation of a wide range of radiotracers [33]. As this methodology has been taken up for the labelling of various precursors, a number of factors have been shown to influence this complex catalytic process. Among them are the sensitivity of the Cu-mediated process to the basic conditions during PTC-controlled solubilisation of [^18^F]fluoride, reaction solvents used, precursor/copper catalyst ratio and others [30,37,38,39].

The feasibility of ^18^F-fluorodeboronation for the synthesis of 6-[^18^F]FP was previously examined using two labeling precursors—commercially available derivatives of boronic acid—6-formylbenzo[*d*][1,3]dioxol-5-yl)boronic acid (**2a**, Figure 2) and pinacolboronate 6-(4,4,5,5-tetramethyl-1,3,2-dioxaborolan-2-yl)benzo[*d*][1,3]dioxole-5-carbaldehyde (**2b**, Figure 2) [29] through application of the so-called “alcohol-enhanced” radiofluorination method [37]. After elution of [^18^F]F^−^ from the quaternary methyl ammonium (QMA) resin with a solution of Et_4_NHCO_3_ in MeOH and evaporation of MeOH, a solution of precursor and Cu(py)_4_(OTf)_2_ in DMA/*n*-BuOH was added to [^18^F]Et_4_NF, followed by heating. The beneficial effect of alcohols as co-solvent in the Cu-mediated process was confirmed for various ArylBPIn substrates [40]. However, according to [29], the attempted Cu-mediated radiofluorination of **2a** and **2b** in DMA/*n*-BuOH 7/3 (100 °C, 10–20 min, Et_4_NHCO_3_ as PTC) did not result in the formation of 6-[^18^F]FP.

Using the two labelling precursors—**2a** and **2b**—we applied our previously developed [^18^F]fluoride trapping-elution protocol by replacing Et_4_NHCO_3_ [29] with the non-basic Bu_4_NOTf (10 μmol) in conjunction with 2-PrOH (0.6 mL) as the eluting solvent [41,42]. The fluoride-PTC complex was eluted directly into the reaction vial containing the labelling precursor and Cu(py)_4_(OTf)_2_ in a suitable solvent (0.8 mL), avoiding any evaporation steps. Optimisation of radiofluorination parameters in terms of the solvents used and amounts of the reactants was realized using commercially available boronic acid derivative **2a** (Figure 2). From several solvents investigated (Table 1), the highest fluorination (RCC of 96 ± 2%, Table 1, Entry 5) was achieved carrying out radiofluorination in the mixture of 2-PrOH/CH_3_CN (2/3) at precursor-to-copper catalyst ratio of 20/20 μmol. This protocol worked equally for radiofluorination of ArylBPin precursor **2b,** providing the desired 6-[^18^F]FP with RCC of >97%. For practical reasons, the use of **2a** as a commercially available precursor would of course be preferable. Further reduction in the reactants amounts down to 10/10 μmol has resulted in ca. 50% decrease in the yield of radiofluorination of **2a** (Table 1, Entry 6). No product formation could be observed when radiofluorination of **2a** was carried out in DMA, a solvent typically used in Cu-mediated radiofluorinations, with 2-PrOH as co-solvent (Table 1, Entry 1). The relatively low conversion rates of **2a** (Table 1, Entries 3, 4) were accompanied by substantial losses of radioactivity on the inner surfaces of the reaction vessel (up to 70% of total radioactivity in the case of neat 2-PrOH) and substantial RCC variability. This could be explained by poor solubility of the labelling precursor in the solvents used.

### 2.3. Synthesis of 6-[^18^F]FP via Diaryliodonium Salts Precursors (Methods C and D)

Another effective approach to the incorporation of [^18^F]fluoride into (hetero)aromatic substrates is the use diaryliodonium (DAI) salt precursors—route pioneered by the Pike group [43]. A very practical approach for performing radiofluorination has been introduced for the onium and DAI salts precursors [44]. The advantageous feature of this approach is that the [^18^F]fluoride retained on the anion-exchange resin is eluted directly with the solution of the DAI precursor in MeOH; following quick removal, the solvent is then directly followed by a fluorination reaction. This type of procedure uses neither the PTC/base nor other additives, it reduces the number of operational steps, saves time and is compatible with base-sensitive precursors and products.

For the synthesis of 6-[^18^F]FP, two types of iodonium salts precursors were investigated (Figure 2): (piperonyl)(mesityl)iodonium *p*-toluenesulfonate (**3**), and (piperonyl)(phenyl)iodonium *p*-toluenesulfonate (**4a**) and bromide (**4b**).

A copper-mediated radiofluorination of (mesityl)(aryl)iodonium (MAI) salts using the commercially available (CH_3_CN)_4_CuOTf complex and [^18^F]KF/18-crown-6 for the activation of the fluorine-18 was suggested in 2014 [45] as an effective route for radiofluorination of the electron rich arenes. It was shown that when involving the use of a bulky mesityl group as an auxiliary force, the nucleophilic substitution acted towards the less sterically hindered site on the arene ring [45]. A recently developed protocol has been applied for the synthesis radiotracers using MAI salts as labelling precursors [46]. In brief, [^18^F]fluoride is eluted from the anion-exchange matrix with solution of MAI precursor in MeOH/DMF (20% MeOH), followed by Cu-mediated fluorination in the same solvent mixture. Despite the high [^18^F]F^−^ elution and fluorination efficiency achieved for a series of ^18^F-fluorinated aromatic amino acids [46], utilising this approach for the preparation of 6-[^18^F]FP from MAI salt **3** (Figure 2) resulted in elution efficiency from anion-exchange matrix using **3** (20 µmol) in 20% MeOH/DMF (0.72 mL) being low, as was the radiofluorination reaction yield (Table 2, Entry 1). Eluting [^18^F]F^−^ with solution of **3** (20 µmol) in pure MeOH (1 mL) provided over 90% [^18^F]F^−^ elution efficiency; however, heating for the purpose of MeOH removal resulted in a complete decomposition of the labelling precursor **3**. As a result, no product was formed (Table 2, Entry 2).

Consequently, we moved on to the more conventional approach for nucleophilic radiofluorination using tetraalkylammonium salts as PTCs for the activation of [^18^F]fluoride. Radionuclide was eluted from the cartridge using 4 µmol of Et_4_NHCO_3_ or Bu_4_NOTs in 1 mL of MeOH followed by the solvent evaporation. The reactive [^18^F]fluoride thus obtained was allowed to react with **3** in the presence of the Cu(I)-catalyst in DMF, affording 6-[^18^F]FP in the RCC of ca. 40–50% when high amounts of the reactants were used (Table 2, Entries 4 and 6). However, despite the improvement in radiofluorination efficiency, the radioactivity yield was not high enough considering the desired 6-[^18^F]FP is an intermediate in a multistep synthesis.

A second strategy which does not involve transition metal catalysts is the radiofluorination of the (phenyl)iodonium salts precursors **4a** and **4b** with different counter ions (Figure 2). Starting with the tosylate salt **4a,** we failed to produce 6-[^18^F]FP consistently using either approach described earlier (Table 2, Entries 7–10) or employing tetralkylammoinum salt for the activation of [^18^F]fluoride (Table 2, Entries 11–14). Gratifyingly, we were able to reach high RCC of 84 ± 5% (*n* = 4) (Supplementary Materials, Figure S18) in radiofluorination of bromide iodonium salt **4b** (Table 2, Entry 16) when performing radiofluorination with two-step heating sequence. Introduction of such a heating procedure was based on our UV-HPLC analysis of samples of the reaction mixture that revealed decomposition of **4b** during the MeOH evaporation step (Table 2, Entry 15). To prevent precursor decomposition, MeOH was removed during the first heating step at 75–85 °C under gentle agitation with nitrogen gas flow, while the radiofluorination step was performed at 120 °C for 20 min in a neat propylene carbonate (PC), affording 6-[^18^F]FP in high RCC. However, using the same protocol, only around 3% RCC was observed in the radiofluorination of the tosylate salt **4a** (Table 2, Entry 14). Such a low radiofluorination efficiency of **4a**, as compared to the bromide salt **4b**, is not supported by the literature data demonstrating that tosylate is one of the most reactive salts towards the [^18^F]fluoride ion [47]. We assume that the radiofluorination might be suppressed by the presence of residual silver in **4a** that was prepared from **4b** by reaction with silver tosylate.

Finally, from all of the investigated radiolabeling procedures, highest RCCs for 6-[^18^F]FP were achieved with Cu-mediated radiofluorination of commercially available **2a** using [^18^F]Bu_4_NOF in 2-PrOH/CH_3_CN (Table 1, Entry 5). However, the radiofluorination of (phenyl)iodonium salt **4b** in MeOH/PC (Table 2, Entry 15) avoiding Cu-catalyst turned out to be most suitable route for preparing 6-[^18^F]FP with additional benefits for the synthesis automation through the use of a fairly simple and straightforward trapping/elution protocol for [^18^F]F^−^ (elution efficiency was over 85%).

### 2.4. Radiosynthesis of [^18^F]Anle138b

**Scheme 2:**
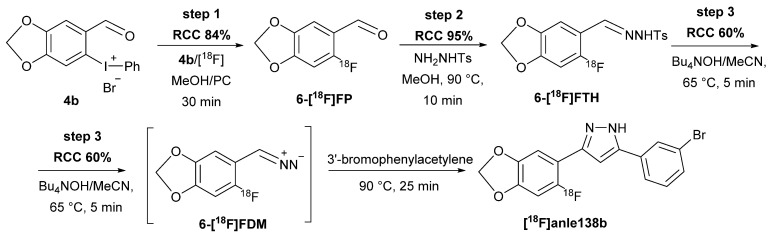
Radiosynthesis scheme for [^18^F]anle138b. Conditions—step 1: Elution of [^18^F]F^−^ with 20 µmol **4b** in 44% MeOH/PC (0.9 mL). Heating for 85 °C, 10 min, then 120 °C, 20 min; step 2: NH_2_NHTs (40 µmol) in MeOH (1 mL). Heating for 90 °C, 10 min under stirring by N_2_ flow; step 3: Bu_4_NOH (26 µmol in 0.4 mL CH_3_CN, 65 °C, 5 min), then 3′-bromophenylacetylene (0.4 mmol in 0.4 mL CH_3_CN, 90 °C, 25 min).

Radiolabeling of [^18^F]anle138b starting from **4b** as a labelling precursor is depicted in Scheme 2.

**Figure 3:**
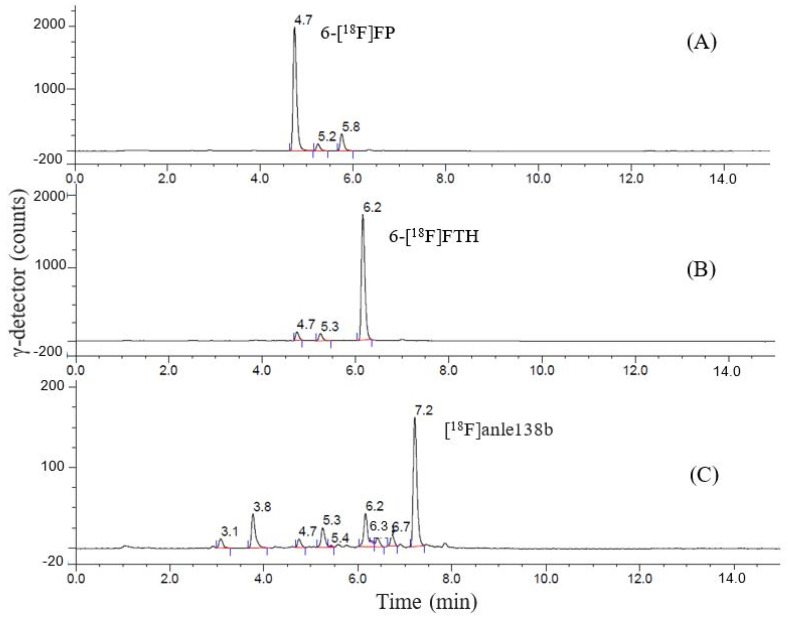
Analytical radioHPLC chromatograms of the reaction mixture (**A**,**B**) and final product (**C**) in the synthesis of [^18^F]anle138b. (**A**) 6-[^18^F]FP; (**B**) 6-[^18^F]FTH; and (**C**) [^18^F]anle138b. HPLC column X-Bridge C18, 150 × 4.6 mm (Waters), gradient of 0.1%TFA/CH_3_CN, and flow rate of 2.0 mL/min.

When developing our method for [^18^F]anle138b, we were, in a significant part, guided by the previously outlined synthetic strategy [29], while focusing our attention on making the synthesis as simple as possible by eliminating intermediate purification steps and employing the one-pot approach. The [^18^F]fluoride isolation and synthesis itself were conducted in a custom built synthesis module, described in the experimental part. For monitoring of radiolabelling progress, aliquots of the relevant reaction mixture were taken after each synthesis step (Scheme 2) and analyzed by radio-HPLC (Figure 3).

As described above, the first synthesis step—the radiofluorination of aryliodonium bromide **4b** afforded 6-[^18^F]FP in the RCC of ca. 84%. Non-isolated 6-[^18^F]FP (solution in MeOH/PC) was used directly in the tosyl hydrazide condensation step (40 µmol of hydrazide in 1 mL of MeOH) at 90 °C for 10 min (step 2, Scheme 2). The reaction proceeded with almost quantitative conversion to 6-[^18^F]fluoro-3,4-methylendioxybenzylidene tosylhydrazone (6-[^18^F]FTH) (Figure 3B). The 6-[^18^F]FTH obtained was converted in situ into the corresponding 6-[^18^F]fluorophenyldiazomethane (6-[^18^F]FDM) by addition of Bu_4_NOH in CH_3_CN and heating to 65 °C for 5 min. Finally, the cycloaddition reaction of 6-[^18^F]FDM with 3′-bromophenylacetylene afforded crude [^18^F]anle138b. Remarkably, the efficiency of the cycloaddition reaction greatly depends on the nature of base employed in the previous step. From several bases investigated (DBU, NaOH, K_2_CO_3_ and LiO*t*Bu) [29], the highest conversion rate of ca. 50% was achieved using LiO*t*Bu. Unfortunately, LiO*t*Bu is insoluble in most organic solvents, including PC that was an essential component of the reaction mixture in our one-pot three-step synthesis procedure. To address this issue, Bu_4_NOH soluble in both CH_3_CN and PC was used as a base. Furthermore, according to the data previously reported [48], the efficiency of one-pot cycloaddition process could be improved by sequential introduction of the base and corresponding acetylene into reaction mixture. Taking those considerations into account, we adjusted our procedure to employ sequential addition of reagents in the following order: The solution of Bu_4_OH in CH_3_CN was added first with heating at 65 °C for 5 min, followed by 3′-bromophenylacetylene addition and a second round of heating at 90 °C for 25 min. With this approach, we were able to obtain [^18^F]anle138b in the RCC of 60% (Figure 3C) using 0.4 mmol 3′-bromophenylacetylene and 26 µmol Bu_4_NOH.

**Scheme 3:**
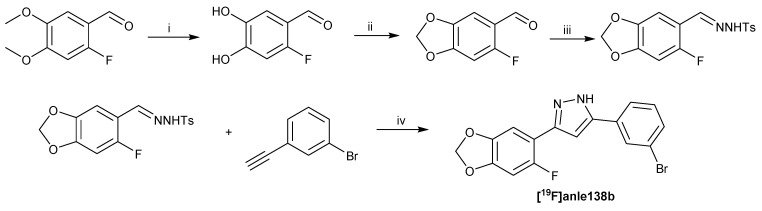
Synthesis of [^19^F]anle138b. Reagents and conditions: (i) BBr_3_, DCM, −80–20 °C, 30 h; (ii) CH_2_Br_2_, K_2_CO_3_, DMF, 110 °C, 5 h; (iii) TsNHNH_2_, CH_3_OH, 20 °C, 4 h; and (iv) LiO*t*Bu, CH_3_CN, reflux, 23 h.

For the identification of the target product, the authentic reference standard [^19^F]anle138b was prepared (Scheme 3); the synthesis is described in the experimental part.

### 2.5. Purification and Quality Control of [^18^F]Anle138b

Due to high lipophilicity of anle138b, isolation of the ^18^F-fluorinated derivative by conventional semi-preparative HPLC presents a challenge. Given the “one-pot” synthesis approach and the resulting multicomponent reaction mixture, the purification task becomes even more complicated.

The isolation of [^18^F]anle138b from the reaction mixture for two different HPLC systems was evaluated. The first one (System A) is an integral part of the GE Tracerlab FX C Pro module, equipped with UV- and β-radioactivity flow detectors. The HPLC separation was performed on a reverse-phase Ascentis RP-Amide semi-preparative column, 250 × 10 mm (Sigma-Aldrich GmbH, Steinheim, Germany). The content of the reaction vessel (1.2 mL) was diluted with the mobile phase and transferred to the 2 mL injection loop. For practical reasons, biocompatible ethanol-containing eluent would be preferable. However, when using H_2_O/EtOH gradient system (gradient conditions 1, Materials and Methods) at a flow rate of 3.0 mL/min, only 5% of the injected radioactivity was recovered as [^18^F]anle138b (R_t_ of 25–26 min) with radiochemical purity (RCP) in order of 80%. Replacing EtOH with CH_3_CN in the mobile phase and modifying gradient (gradient conditions 2, Materials and Methods) afforded [^18^F]anle138b in 90% RCP with slightly increased recovery of the radioactivity (8%) in the product fraction (R_t_ of 28–32 min).

**Figure 4:**
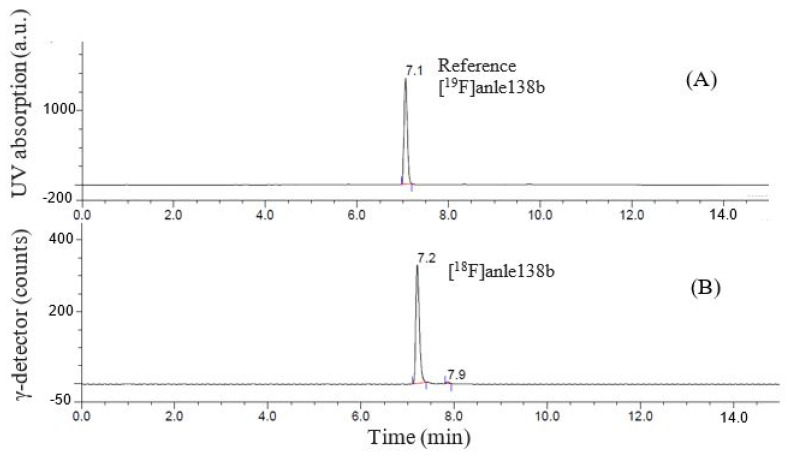
HPLC analysis of the formulated [^18^F]anle138b. Conditions: HPLC column X-Bridge C18, 150 × 4.6 mm (Waters Corporation (Millford, CT, USA), gradient of 0.1%TFA/CH_3_CN with a flow rate 2.0 mL/min, UV is254 nm: (**A**) reference standard [^19^F]anle138b; (**B**) the formulated [^18^F]anle138b.

Alternatively, we investigated applicability of a different HPLC column, Chromolith SemiPrep RP-18e, 100 × 10 mm equipped with a UV and radioactivity detector, a gradient pump and the Rheodyne-type injector with a 100 μL loop (System B). Under gradient conditions using EtOH-based mobile phase at a flow rate of 4 mL/min, the [^18^F]anle138b (fraction with R_t_ 9.7–9.9 min, 1 mL volume, 26% recovery of the product) was obtained in more than 98% RCP according to radioHPLC (Figure 4), and ca. 100% according to radioTLC (Supplementary Materials, Figure S19) As the ethanol-containing eluent was applied, cartridge based reformulation of the product was not required.

The main limitation of this method is a small maximum injection volume (100 µL); with higher injected volumes, the column performance would degrade due to overloading. Therefore, multiple injections would be required for purification of all reaction volume produced in the synthesis (1.2 mL total). Nonetheless, the procedure developed is well suited for preclinical studies where small amounts of the radiotracer are typically injected.

Recalculating the radioactivity of the volume injected (100 μL) to total reaction volume (1.2 mL), decay-corrected RCY of [^18^F]anle138b was 15.1 ± 2.3% (*n* = 3) with synthesis time of ca. 105 min. *A*_m_ was in the range of 31.5–79.5 GBq/µmol.

## 3. Materials and Methods

### 3.1. General Chemistry

All commercially available chemicals were used without any further purification. Analytical thin-layer chromatography (TLC) on Merck 60 F254 silica gel plates with UV visualization, 254 nm (Merck KGaA, Darmstadt, Germany) was performed. Column chromatography has been carried out on silica gel 60 (0.035–0.070 mm (Acros Organics, Geel, Belgium) with the indicated eluents. NMR spectra were recorded on a Bruker Avance 400 spectrometer (Bruker Optik GmbH, Ettlingen, Germany) in CDCl_3_ or DMSO-d6 (^1^H, ^13^C and ^19^F at 400.17, 100.62 and 376.54 MHz, respectively). HRMS (ESI) analysis was done on a Bruker micrOTOF mass spectrometer (Bruker Optik GmbH, Ettlingen, Germany).

#### 3.1.1. The 4 Steps Synthesis of 3-(6-Fluoro-1,3-benzodioxol-5-yl)-5-(3-bromophenyl)-1H-pyrazole ([^19^F]Anle138b) (Scheme 3)

##### 2-Fluoro-4,5-dihydroxybenzaldehyde [49]

Under inert atmosphere, 6-fluoroveratraldehyde (1.00 g, 5.43 mmol) was dissolved in anhydrous DCM (10 mL). The mixture was cooled near −80 °C and then boron tribromide (4.4 g, ~17 mmol) was added dropwise for 5 min. The reaction mixture was allowed to warm to room temperature and stirred for 30 h. The red–violet mixture was poured on the ice (150 g) and extracted with ethyl acetate (3 × 20 mL). After drying with MgSO_4_ and concentrating under reduced pressure, the residue was purified by column chromatography on silica gel (heptane/ethyl acetate: 1/1) to afford 2-fluoro-4,5-dihydroxybenzaldehyde (0.65 g, yield of 77%) as dark solid. NMR spectra have been shown in the Supplementary Materials. ^1^H-NMR (400 MHz, DMSO-*d*_6_) δ 6.65 (d, ^3^*J*_HF_ = 11.9 Hz, 1H, C^3^H); 7.10 (d, ^4^*J*_HF_ = 6.9 Hz, 1H, C^6^H); 9.59 (br s, OH); 9.98 (s, 1H, CHO); 10.69 (bs, OH). ^13^C-NMR (101 MHz, DMSO-*d*_6_) δ 103.05 (d, *J*_CF_ =24.5 Hz); 112.23; 115.24 (d, *J*_CF_ =8.9 Hz); 142.64; 153.82; 159.26 (d, ^1^*J*_CF_ =249.4 Hz, C^2^); 185.61 (d, ^3^*J*_CF_ =5.7 Hz, CHO).

##### 6-Fluorobenzo-1,3-dioxole-5-carbaldehyde [50]

Under inert atmosphere, the mixture of 2-fluoro-4,5-dihydroxybenzaldehyde (0.65 g, 4.16 mmol), dibromomethane (1.50 g, 8.62 mmol) and K_2_CO_3_ (1.38 g, 10.00 mmol) in DMF (10 mL) was stirred at 110 °C for 5 h. After cooling, ethyl acetate (10 mL) was added, and mixture was filtered. The solid was washed with ethyl acetate (20 mL). The liquid was concentrating under reduced pressure to the volume of ~2 mL. The residue was purified by column chromatography on silica gel (heptane/ethyl acetate: 1/1) to afford 6-fluorobenzo-1,3-dioxole-5-carbaldehyde (0.35 g, yield of 50%) as yellow solid. NMR spectra have been shown in the Supplementary Materials. ^1^H-NMR (400 MHz, CDCl_3_) δ 6.04 (s, 2H, OCH_2_O); 6.60 (d, ^3^*J*_HF_ = 9.7 Hz, 1H, C^7^H); 7.16 (d, ^4^*J*_HF_ = 5.6 Hz, 1H, C^4^H); 10.12 (s, 1H, CHO). ^13^C-NMR (101 MHz, CDCl_3_) δ 97.97 (d, *J*_CF_ =28.9 Hz); 102.97 (OCH_2_O); 104.94 (d, *J*_CF_ =3.0 Hz); 117.87 (d, *J*_CF_ =9.0 Hz); 144.87; 154.09 (d, *J*_CF_ =14.8 Hz); 162.60 (d, ^1^*J*_CF_ =254.0 Hz, C^6^); 185.48 (d, ^3^*J*_CF_ =8.5 Hz, CHO). ^19^F-NMR (376.5 MHz, CDCl_3_) δ -126.1.

##### N′-((6-Fluorobenzo-1,3-dioxole-5-yl)methylene)-4-methylbenzenesulfonohydrazide

A mixture of 6-fluorobenzo-1,3-dioxole-5-carbaldehyde (0.45 g, 2.68 mmol) and *p*-toluenesulfonyl hydrazide (0.55 g, 2.95 mmol) in methanol (10 mL) was stirred at room temperature for 4 h. Yellow precipitate was filtered and washed with MeOH (2 × 2 mL) to afford the title compound (0.50 g, yield of 56%) as a grey solid. NMR spectra have been shown in the Supplementary Materials. ^1^H-NMR (400 MHz, DMSO-*d*_6_) δ 2.36 (s, 3H, CH_3_); 6.10 (s, 2H, OCH_2_O); 6.98 (d, ^3^*J*_HF_ = 10.0 Hz, 1H, C^7^H); 7.05 (d, ^4^*J*_HF_ = 6.0 Hz, 1H, C^4^H); 7.41 (d, *J* = 8.0 Hz, 2H); 7.76 (d, *J* = 8.0 Hz, 2H); 7.97 (s, 1H); 11.44 (br s, NH). ^13^C-NMR (101 MHz, DMSO-*d*_6_) δ 21.10; 98.20 (d, *J*_CF_ = 30.0 Hz); 102.71 (OCH_2_O); 113.64 (d, *J*_CF_ = 11.8 Hz); 127.35; 129.81; 135.93; 139.90; 139.95; 143.69; 144.52; 150.14 (d, *J*_CF_ =15.1 Hz); 156.58 (d, ^1^*J*_CF_ =244.0 Hz, C^6^). ^19^F-NMR (376.5 MHz, DMSO-*d*_6_) δ -126.4.

##### (6-Fluoro-1,3-benzodioxol-5-yl)-5-(3-bromophenyl)-1H-pyrazole ([^19^F]Anle138b)

Under inert atmosphere, lithium tert-butoxide (0.22 g, 2.76 mmol) was added into a solution of *N*′-((6-fluorobenzo-1,3-dioxole-5-yl)methylene)-4-methylbenzenesulfonohydrazide (0.34 g, 1.01 mmol) in dry acetonitrile (10 mL) and stirred at room temperature for 15 min. Then, 3-bromophenylacetylene (0.50 g, 2.76 mmol) was added and the reaction was refluxed for 23 h. The mixture was evaporated in vacuo, and water (10 mL) was added. After extraction with ethyl acetate (2 × 20 mL), the organic phase was concentrated under reduced pressure. The residue was purified by column chromatography on silica gel (heptane/ ethyl acetate: 1/1) to afford [^19^F]anle138b (0.10 g, yield of 28%) as a grey solid. ^1^H-NMR (400 MHz, CDCl_3_) δ 6.05 (s, 2H, OCH_2_O); 6.72 (d, *J* = 10.6 Hz, 1H); 6.82 (s, 1H); 7.13 (br d, *J* = 6.0 Hz, 1H); 7.30 (t, *J* = 8.0 Hz, 1H); 7.47 (m, 1H); 7.74 (br d, *J* = 8.0 Hz, 1H); 7.97 (br s, 1H); 10.61 (br s, NH). ^19^F-NMR (376.5 MHz, CDCl_3_) δ -121.4. HRMS (ESI): *m*/*z* [M + H]^+^ calculated for C_16_H_10_BrFN_2_O_2_: 360.9982; found: 360.9985. This process involved correct isotopic pattern.

#### 3.1.2. (6-Formylbenzo-1,3-dioxole-5-yl)(phenyl)iodonium Bromide (**4b**) (Figure 2) [44]

Boron trifluoride etherate (0.74 g, 5.21 mmol) was added dropwise to an ice cold solution of 2-formyl-4,5-methylenedioxyphenylboronic acid (0.50 g, 2.58 mmol) in anhydrous DCM (15 mL) under Ar. The resulting yellow suspension was stirred for 30 min at 0 °C. (Diacetoxyiodo)benzene (1.25 g, 3.87 mmol) was added, and the mixture was stirred for 1 h at 20 °C. The solvent was removed under reduced pressure. The residue was taken into saturated NaBr-H_2_O (15 mL) for 10 min. The resulting yellow suspension was filtered and washed with a H_2_O/CH_3_CN mixture (8/1, 10 mL). The solid was refluxed in DCM (10 mL). Insoluble material was filtered off and dried in vacuo to afford **4b** (0.95 g, yield of 85%) as grey solid. ^1^H-NMR (400 MHz, DMSO-*d*_6_) δ 6.27 (s, 2H, OCH_2_O); 7.27 (s, 1H); 7.56 (t, *J* = 7.8 Hz, 2H); 7.68–7.76 (m, 1H); 7.72 (s, 1H); 8.24 (d, *J* = 7.8 Hz, 2H); 10.06 (s, 1H, CHO). ^13^C-NMR (101 MHz, DMSO-*d*_6_) δ 104.20; 112.65; 113.13; 114.04; 116.93; 127.72; 131.76; 132.21; 136.01; 150.27; 154.44; 192.43.

#### 3.1.3. (6-Formylbenzo-1,3-dioxole-5-yl)(phenyl)iodonium Tosylate (**4a**) (Figure 2)

The solution of TsOAg (0.42 g, 1.50 mmol) in MeOH/MeCN mixture (1/3; 8 mL) was added to the suspension of compound **4b** (0.60 g, 1.38 mmol) in MeOH/MeCN mixture (1/1; 10 mL) at 20 °C. After stirring for1 h, the suspension was filtered, and the resulting solution was evaporated under reduced pressure. The residue was refluxed in DCM (10 mL) and filtered (7 times). Insoluble material was dried in vacuo to afford **4a** (0.23 g, yield of 32%) as grey solid. ^1^H-NMR (400 MHz, DMSO-*d*_6_) δ 2.28 (s, 3H); 6.30 (s, 2H, OCH_2_O); 7.07 (s, 1H); 7.10 (d, *J* = 7.9 Hz, 2H, TsO); 7.46 (d, *J* = 7.9 Hz, 2H, TsO); 7.65 (t, *J* = 7.8 Hz, 2H); 7.81 (m, 1H); 7.83 (s, 1H); 8.28 (dd, *J* = 8.2; 1.0 Hz, 2H); 10.06 (s, 1H, CHO). ^13^C-NMR (101 MHz, DMSO-*d*_6_) δ 20.80; 104.54; 107.36; 113.23; 113.76; 114.25; 125.49; 127.53; 128.05; 132.19; 133.07; 136.57; 137.60; 145.75; 150.61; 154.96; 192.70.

#### 3.1.4. (6-Formylbenzo-1,3-dioxole-5-yl)(2,4,6-trimethylphenyl)iodonium Bromide

This compound was prepared analogously to compound **4b** using 2,4,6-trimethyl(diacetoxyiodo)benzene; the crude solid was refluxed in acetone (10 mL) instead of DCM for purification. 6-formylbenzo-1,3-dioxole-5-yl)(2,4,6-trimethylphenyl)iodonium bromide (0.65 g, yield of 66%) was obtained as a grey–yellow solid. ^1^H-NMR (400 MHz, CDCl_3_) δ 2.38 (s, 3H, CH_3_); 2.58 (s, 6H, 2CH_3_); 6.14 (s, 1H); 6.15 (s, 2H, OCH_2_O); 7.14 (s, 2H); 7.57 (s, 1H); 10.02 (s, 1H, CHO). ^13^C-NMR (101 MHz, CDCl_3_) δ 21.16; 26.91; 103.90; 109.77; 112.17; 115.88; 121.21; 127.89; 129.97; 141.96; 143.84; 149.98; 155.99; 191.45.

#### 3.1.5. (6-Formylbenzo-1,3-dioxole-5-yl)(2,4,6-trimethylphenyl)iodonium Tosylate (**3**) (Figure 2)

The solution of TsOAg (0.23 g, 0.82 mmol) in MeOH/MeCN mixture (3/1; 4 mL) was added to the solution of compound (6-formylbenzo-1,3-dioxole-5-yl)(2,4,6-trimethylphenyl)iodonium bromide (0.36 g, 0.75 mmol) in a MeOH/MeCN mixture (5/1; 6 mL) at 20 °C. After stirring for 1 h, the suspension was filtered and resulted solution was evaporated under reduced pressure. The residue was refluxed in DCM (10 mL) and filtered. The solution was evaporated under reduced pressure to afford **3** (0.23 g, yield of 54%) as a grey solid. ^1^H-NMR (400 MHz, CDCl_3_) δ 2.29 (s, 3H); 2.37 (s, 3H); 2.50 (s, 6H, 2CH_3_); 6.14 (s, 1H); 6.16 (s, 2H, OCH_2_O); 6.98 (d, *J* = 7.7 Hz, 2H, TsO); 7.05 (s, 2H); 7.43 (d, *J* = 7.7 Hz, 2H, TsO); 7.71 (s, 1H); 10.16 (s, 1H, CHO). ^13^C-NMR (101 MHz, CDCl_3_) δ 21.41; 21.42; 26.93; 104.22; 104.73; 109.35; 116.62; 118.38; 125.94; 127.98; 128.37; 130.08; 139.05; 143.03; 143.46; 144.55; 150.78; 156.35; 192.09.3.5.

### 3.2. Radiochemistry

#### 3.2.1. General

Unless otherwise stated, reagents and solvents were commercially available and were used without further purification. Anhydrous acid free MeCN (max 10 ppm H_2_O) was purchased from Kriochrom, St. Petersburg, Russia. The precursors **3**, **4a**, **4b**, reference standard 6-fluorobenzo-1,3-dioxole-5-carbaldehyde (6-[^19^F]FP) and reference standard [^19^F]anle138b were prepared as described above. The 6-formylbenzo[*d*][1,3]dioxol-5-yl)boronic acid (**2a**) was obtained from Sigma-Aldrich GmbH (Steinheim, Germany). Cu(MeCN)_4_OTf and Cu(OTf)_2_(py)_4_ were obtained from Sigma-Aldrich GmbH (Steinheim, Germany) and stored under argon. Deionized water (18.2 MOhm*cm) from an in-house Millipore Simplicity purification system (Merck KGaA, Darmstadt, Germany) was used for the preparation of all aqueous solutions. [^18^O]H_2_O (97% enrichment) was purchased from Global Scientific Technologies, Sosnovyi Bor, Russia. Sep-Pak Accell Plus QMA Plus Light Cartridges (130 mg) were acquired from Waters Corporation (Millford, CT, USA) and were conditioned with 10 mL of 0.05 M NaHCO_3_, followed by 10 mL of H_2_O before application.

Radio-TLC analyses were carried out on silica gel plates (60 F254, Merck or Sorbfil, Lenchrom, Russia); radioactivity distribution was determined using a Scan-RAM radioTLC scanner controlled by the chromatography software package Laura (v6.0.4.92) for PET (LabLogic, Sheffield, UK). An aliquot (2–3 µL) of the crude reaction mixture diluted with acetonitrile, was applied onto a TLC plate, and the plate was then developed in ethyl acetate. The R_f_ values for [^18^F]fluoride, 6-[^18^F]FP and [^18^F]anle138b were 0.05, 0.57 and 0.67, correspondingly. The radiochemical conversion (RCC) measured by radioTLC was defined as the ratio of the product peak area to the total peak area on the TLC. RCC values were not corrected for radioactive decay.

Analytical HPLC was performed on a Dionex ISC-5000 system (Dionex, Sunnyvale, CA, USA). It was equipped with a gradient pump, Rheodyne type injector with a 20 μL loop and a UV absorbance detector with variable wavelength (set to 254 nm) connected in series with a radiodetector (Carrol and Ramsey Associates, CA, USA, model 105-S) giving a delay of 0.1 min. The identity, radiochemical and chemical purity of the [^18^F]anle138b and analysis of the reaction mixture at each stage of the synthesis were determined under the following HPLC conditions: X-Bridge C18 HPLC column, 150 × 4.6 mm (Waters Corporation, Millford, CT, USA), eluent with 5–95% gradient (0.1% aq. TFA/MeCN), and a flow rate of 2.0 mL/min. Overall, there was a 0–8.0 min 5–95% MeCN linear increase; 8.0–11.0 min 95% MeCN isocratic; 11.0–11.2 min 95–5% MeCN linear decrease; and an 11.2–15.0 min 5% MeCN isocratic. The R_t_ values for the precursor, reference and radiolabelled intermediates are presented in Table 3.

For the isolation of [^18^F]anle138b, two different HPLC systems were used.

System A employed an HPLC package available on GE Tracerlab FX C Pro module (GE Healthcare, Waukesha, WI, USA), consisting of a SYCAM S1122 pump, UV detector KNF (λ = 254 nm), LAB LABOPORT, 2 mL injection loop and a β-radioactivity detector. Conditions for this system consisted of a column Ascentis RP-AMIDE, 250 × 10 mm, 5 µm (Supelco, Bellefonte, PA, USA). For gradient 1 (H_2_O /EtOH), the flow rate was 3.0 mL/min with 0–10.0 min 60% EtOH, 10.0–30.0 min 80% EtOH; gradient 2: (H_2_O/CH_3_CN), flow rate of 4.5 mL/min with 0–27.0 min 55% CH_3_CN; 27.0–35.0 min CH_3_CN.

System B employed Dionex ISC-5000 HPLC system (Dionex, Sunnyvale, CA, USA), described above, with a 100 μL injection loop and Chromolith SemiPrep RP-18e column, 100 × 10 mm (Merck KGaA, Darmstadt, Germany). Conditions: 5 to 95% gradient (H_2_O/EtOH) with a flow rate of 4.0 mL/min; 0–10.0 min 5–95% EtOH linear increase; 10.0–12.0 min 95% EtOH isocratic; 12.0–12.1 min 95–5% EtOH linear decrease; and 12.1–16.0 5% EtOH isocratic.

#### 3.2.2. Production of [^18^F]Fluoride

[^18^F]Fluoride was produced via the ^18^O(p,n)^18^F nuclear reaction by irradiation of [^18^O]H_2_O (97% enrichment, Global Scientific Technologies, Sosnovyj Bor, Russia) in a niobium target (1.4 mL) with 16.4 MeV protons at a PETtrace 4 cyclotron (GE Healthcare, Uppsala, Sweden). The irradiated [^18^O]H_2_O was transferred from the target using a flow of helium into the collection vial.

#### 3.2.3. Synthesis of 6-[^18^F]Fluoropiperonal (6-[^18^F]FP)

Method B (Figure 2). Cu-mediated radiofluorination of pinacol arylboronates (**2a**, **2b**).

**Figure 5:**
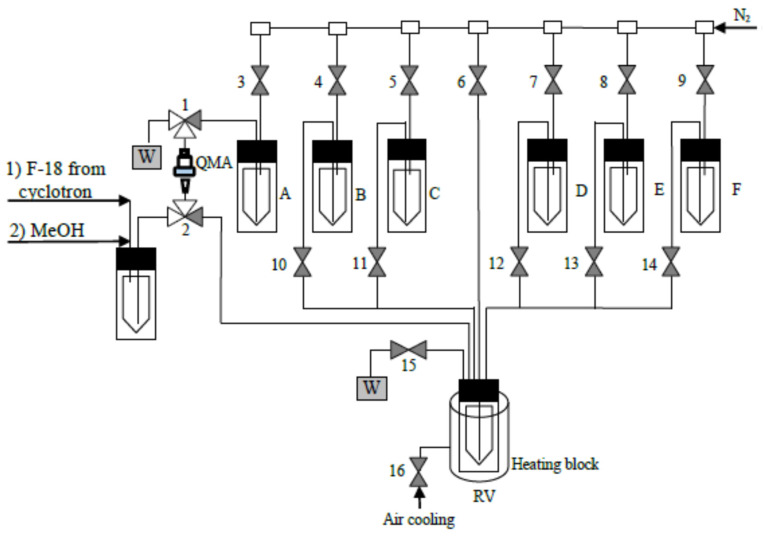
Process flow diagram (PFD) for the semi-automated radiosynthesis of [^18^F]anle138b. (A) a solution of precursor **4b** (20 µmol) in 44% MeOH/PC (0.9 mL); (B) a solution of NH_2_NHTs (40 µmol) in MeOH (1 mL); (C) a solution of Bu_4_NOH (26 µmol) in MeCN (0.4 mL); and (D) a solution of 3′-bromophenylacetylene (0.4 mmol) in CH_3_CN (0.4 mL); E and F are not used.

A solution of [^18^F]F^–^ (0.5–1.0 GBq) in [^18^O]H_2_O was loaded from the male side onto a QMA cartridge. The cartridge was flushed from the male side with 2-PrOH (4 mL) and dried with N_2_ gas for 2 min. ^18^F^–^ was eluted from the female side of the cartridge with a solution of Bu_4_NOTf (4 mg, 10 μmol) in 2-PrOH (0.6 mL) into the 2 mL reaction vessel prefilled with a solution of **2a** or **2b** (20 μmol) and Cu(py)_4_(OTf)_2_ (20 μmol) in MeCN (0.8 mL). The reaction mixture was heated at 65 °C for 10 min, followed by a second round of heating 110 °C for 10 min, while the reactor was sealed via valve 16 (Figure 5). Then, the reaction vessel was cooled down to 40 °C.

Method C (Figure 2). Cu-mediated radiofluorination of (piperonyl)(mesityl)iodonium *p*-toluenesulfonate (**3**).

A solution of [^18^F]F^–^ (0.5–1.0 GBq) in [^18^O]H_2_O was loaded from the male side onto a QMA cartridge. The cartridge was flushed from the male side with MeOH (2 mL) and dried with N_2_ gas for 2 min. ^18^F^–^ was eluted from the female side of the cartridge with a solution of Et_4_NHCO_3_ (0.8 mg, 4.2 μmol) in MeOH (1 mL) into the 2 mL reaction vessel. The solvent was evaporated by heating to 75 °C under gas flow, and the reaction vessel was then cooled to 50 °C. The solution of **3** (30 μmol) and Cu(MeCN)_4_OTf (30 μmol) in DMF (0.5 mL) was then added to the dried residue and the reaction mixture, and the reaction mixture was heated at 90 °C for 20 min, while the reactor was sealed via valve 16 (Figure 5). Then, the reaction vessel cooled down to 40 °C.

Method D (Figure 2). Radiofluorination of diaryliodonium salts **4a**, **4b**.

A solution of [^18^F]F^–^ (0.5–1.0 GBq) in [^18^O]H_2_O was loaded from the male side on a QMA cartridge. The cartridge was flushed from the male side with MeOH (2 mL) and dried with N_2_ gas for 2 min. ^18^F^–^ was eluted from the female side of the cartridge with a solution of the respective radiolabelling precursor **4a** or **4b** (20 μmol) in 44% MeOH/PC (0.9 mL). The reaction mixture was heated at 85 °C for 10 min with stirring N_2_ followed by the second round of heating at 120 °C for 20 min, while the reactor was sealed via valve 16 (Figure 5). The reaction vessel was cooled down to 40 °C.

#### 3.2.4. Synthesis of [^18^F]Anle138b from **4b** (Scheme 2)

Step 1. Radiofluorination of diaryliodonium salt **4b** (Method D).

A solution of [^18^F]F^–^ (9.0–11.0 GBq) in [^18^O]H_2_O was loaded onto a QMA cartridge from the male side followed by flushing with MeOH (2 mL) and drying with N_2_ gas for 2 min. ^18^F^–^ was eluted from the female side of the cartridge with a solution of **4b** (20 μmol) in 44% MeOH/PC (0.9 mL). The reaction mixture was heated at 85 °C for 10 min with stirring by nitrogen flow followed by the second round of heating at 120 °C for 20 min, while the reactor was sealed via valve 16 (Figure 5). The reaction vessel was cooled down to 40 °C.

Step 2. Synthesis of 6-[^18^F]fluoro-3,4-methylendioxybenzylidene tosylhydrazone (6-[^18^F]FTH).

The solution of NH_2_NHTs (40 µmol) in MeOH (1 mL) was added to the reaction mixture obtained at step 1; the content was heated for 10 min at 90 °C under stirring by nitrogen flow. The reaction vessel was cooled down to 65 °C.

Step 3. Synthesis of [^18^F]anle138b.

The solution of Bu_4_NOH (26 µmol) in MeCN (0.4 mL) was added to the reaction mixture obtained at step 2 and the content was heated for 5 min at 65 °C. Then the solution of 3′-bromophenylacetylene (0.4 mmol) in MeCN (0.4 mL) was added and the reaction mixture was heated for 25 min at 90 °C. The reaction vessel was cooled down to 40 °C.

#### 3.2.5. HPLC Purification

System A. The content of the reaction vessel (1.2 mL volume) was diluted with 0.8 mL 50% EtOH or with 0.8 mL of CH_3_CN. The resulting solution was transferred to 2 mL HPLC loop. The fraction containing [^18^F]anle138b (R_t_ of 25–26 min under gradient 1 conditions or 28–32 min under gradient 2 conditions) was collected and analysed for radiochemical and chemical purity.

System B. The aliquot (100 µL) of the reaction mixture (from 1.2 mL total volume) was loaded onto 100 µL of HPLC loop. The fraction containing [^18^F]anle138b (R_t_ of 9.7–9.9 min, 1 mL volume) was collected through a 0.22 µm filter (Millipore, Burlington, MA, USA) attached to a vented sterile vial prefilled with the formulation buffer.

### 3.3. Semi-Automated Synthesis of [^18^F]Anle138b (Figure 5)

The [^18^F]fluoride processing and all the synthesis steps were completed in a custom-built synthesis module described in detail elsewhere [46]. The reactions were performed in a 5 mL V-vial (RV, Figure 5) with a screw cap (Wheaton-vials, Sigma-Aldrich GmbH, Steinheim, Germany). Nitrogen gas was applied for the reagents transfer.

1. Loading of [^18^F]fluoride (9.0–11.0 GBq) onto a QMA anion exchange cartridge.
2. Washing of the cartridge with MeOH (2 mL) and drying with N_2_ gas for 2 min.
3. Elution of [^18^F]fluoride from the QMA cartridge with a solution of **4b** (20 µmol) in 44% MeOH/PC (0.9 mL) into reaction vessel (RV).
4. Heating reaction mixture in the RV at 85 °C for 10 min with stirring by N_2_ with valve 16 open; then, valve 16 is closed and the reaction mixture is heated at 120 °C for 20 min.
5. Cooling down RV to 40 °C; valve 16 is open.
6. Addition of NH_2_NHTs (40 µmol) in MeOH (1 mL).
7. Heating reaction mixture at 90 °C for 10 min with stirring by N_2_ gas._._
8. Cooling down RV to 65 °C.
9. Addition of Bu_4_NOH (26 µmol) in MeCN (0.4 mL).
10. Heating reaction mixture at 65 °C for 5 min; valve 16 is closed.
11. Addition of the solution of 3′-bromophenylacetylene (0.4 mmol) in MeCN (0.4 mL) (valve 16 is open).
12. Valve 16 is closed, heating reaction mixture in RV at 90 °C for 25 min.
13. Cooling down RV to 40 °C; valve 16 is open.
14. Loading reaction mixture (100 µL) into HPLC loop (HPLC system B).
15. Isolation of the product using EtOH-H_2_O gradient system with flow rate of 4 mL/min.
16. Manual collection of the product fraction into a vented collection vial.

## 4. Conclusions

In the current paper, we have outlined a novel, but relatively simple, one-pot three-step procedure for the radiosynthesis of [^18^F]anle138b bypassing any intermediate purification steps. Using base- and phase-transfer catalyst-free radiofluorination procedure with diaryliodonium salt precursor (**4b**), 6-[^18^F]fluoropiperonal—the starting building block for the entire [^18^F]anle138b molecule—was obtained with RCC of >85%. Careful optimisation of the conditions for the following condensation and cycloaddition reactions performed without solvent exchange steps allowed to complete synthesis of [^18^F]anle138b within 105 min with RCY of 15 ± 3% (*n* = 3) and with *A*_m_ in the range of 32–80 GBq/µmol, significantly improving upon previously published results in both yield and synthesis time. While there is still space for further optimization, in particular in the area of HPLC purification, the procedure developed is well suited for [^18^F]anle138b production for use in preclinical studies in animal or cell models. In addition, the suggested methodology may find further use in the preparation of other PET imaging agents derived from the pyrazoles backbone.

## Figures and Tables

**Table 1:** Optimization of Cu-mediated radiofluorination of **2a**; elution of [^18^F]F^−^ by 10 μmol Bu_4_NOTf in 2-PrOH (0.6 mL); precursor/catalyst in 0.8 mL of 2-PrOH/solvent; reaction mixture volume of 1.4 mL; ^a^ 110 °C, 20 min; ^b^ 65 °C, 10 min followed by 110 °C, 10 min; RCC—radiochemical conversion of **2a** into 6-[^18^F]FP as determined from radioTLC data.

Entry	2a/Cu(py)_4_(OTf)_2_, µmol	Radiofluorination Conditions	RCC, %
1	10/20	2-PrOH/DMA ^a^	0; 5
2	10/20	2-PrOH/acetone ^a^	13; 26
3	10/20	2-PrOH ^a^	29 ± 20 (*n* = 3)
4	20/20	2-PrOH ^a^	32 ± 22 (*n* = 3)
5	20/20	2-PrOH/CH_3_CN ^b^	96 ± 2 (*n* = 6)
6	10/10	2-PrOH/CH_3_CN ^b^	48 (*n* = 1)
7	15/15	2-PrOH/CH_3_CN ^b^	86 (*n* = 1)
8	20/15	2-PrOH/CH_3_CN ^b^	64 (*n* = 1)

**Table 2:** Synthesis of 6-[^18^F]FP via radiofluorination of diaryliodinium salts **3**, **4a**, and **4b** using various [^18^F]F^−^ sorption/elution and radiofluorination protocols; RCC—radiochemical conversion of **3**, **4a**, and **4b** into 6-[^18^F]FP as determined from radioTLC data.

Entry	Eluent Composition	Evaporation Step	Precursor/µmol	(CH_3_CN)_4_ CuOTf, µmol	Radiofluorination		RCC, %
					Solvent, mL	T, °C/t, min	
1	**3** (20 µmol) in 20% MeOH/DMF (0.72 mL)	-	-	20	MeOH/DMF (0.72)	90/20	10
2	**3** (20 µmol) in MeOH (1 mL)	+	-	20	DMF (0.5)	90/20	0
3	Et_4_NHCO_3_/MeOH (1 mL)	+	**3**/20	20	DMF (0.5)	90/20	35
4	Et_4_NHCO_3_/MeOH (1 mL)	+	**3**/30	30	DMF (0.5)	90/20	43
5	Bu_4_NOTs/MeOH (1 mL)	+	**3**/20	20	DMF (0.5)	90/20	27
6	Bu_4_NOTs/MeOH (1 mL)	+	**3**/30	30	DMF (0.5)	90/20	52
7	**4a** (20 µmol) in 20% MeOH/DMF (0.72 mL)	-	-	-	MeOH/DMF (0.72)	100/20	3
8	**4a** (20 µmol) in 20% MeOH/DMF (0.72 mL)	-	-	-	MeOH/DMF (0.72)	140/20	11
9	**4a** (20 µmol) in MeOH (1 mL)	-	-	-	MeOH/DMSO (1/0.5)	160/15	5
10	**4a** (20 µmol) in MeOH (1 mL)	+	-	-	DMSO (0.5)	160/15	0
11	Et_4_NHCO_3_/ MeOH (1 mL)	+	**4a**/20	-	DMSO (0.5)	130/15	4
12	Et_4_NHCO_3_/ MeOH (1 mL)	+	**4a**/20	-	DMSO (0.5) CH_3_CN (0.5)	160/15	3
13	Et_4_NHCO_3_/ MeOH (1 mL)	+	**4a**/20	-	DMSO (0.5) CH_3_CN (0.5)	80/20	3
14	**4a** (20 µmol) in 44% MeOH/PC (0.9 mL)	-	-	-	MeOH/PC (0.9)	(1) 75–85/10 (2) 120/20	3 ± 1 (*n* = 3)
15	**4b** (20 µmol) in MeOH (0.8 mL)	+	-	-	PC (0.6)	110/15	0
16	**4b** (20 µmol) in 44% MeOH/PC (0.9 mL)	-	-	-	MeOH/PC (0.9)	(1) 75–85/10 (2) 120/20	84 ± 5 (*n* = 6)

**Table 3:** R_t_ values for the precursor **4b**, reference [^19^F]anle138b and radiolabelled intermediates.

Synthesis Step	Compound	Retention Time by γ-Detector, min	Retention Time by UV-254, min
	Precursor **4b**		2.9
Step 1	6-[^18^F]FP	4.7	
	6-[^19^F]FP		4.6
Step 2	6-[^18^F]FTH	6.1	
Step 3	[^18^F]anle138b	7.1	
Reference	[^19^F]anle138b		7.0

## Data Availability

The data presented in this study are available on request from the corresponding author.

## Supplementary Materials

The following supporting information can be downloaded at https://www.mdpi.com/article/10.3390/molecules28062732/s1, Block I: NMR and HRMS (ESI) spectra. Figure S1: ^1^H NMR spectrum for 6-fluorobenzo-1,3-dioxole-5-carbaldehyde, Figure S2: ^13^C NMR spectrum for 6-fluorobenzo-1,3-dioxole-5-carbaldehyde, Figure S3: ^19^F NMR spectrum of 6-fluorobenzo-1,3-dioxole-5-carbaldehyde, Figure S4: ^1^H NMR spectrum of *N*′-((6-fluorobenzo-1,3-dioxole-5-yl)methylene)-4-methylbenzenesulfonohydrazide, Figure S5: ^13^C NMR spectrum of *N*′-((6-fluorobenzo-1,3-dioxole-5-yl)methylene)-4-methylbenzenesulfonohydrazide, Figure S6: ^19^F NMR spectrum of *N*′-((6-fluorobenzo-1,3-dioxole-5-yl)methylene)-4-methylbenzenesulfonohydrazide, Figure S7: ^1^H NMR spectrum of [^19^F]anle138b, Figure S8: ^19^F NMR spectrum of [^19^F]anle138b, Figure S9: HRMS (ESI) spectrum of [^19^F]anle138b, Figure S10: ^1^H NMR spectrum of compound **4b**, Figure S11: ^13^C NMR spectrum of compound **4b**, Figure S12: ^1^H NMR spectrum for compound **4a**, Figure S13: ^13^C NMR spectrum for compound **4a**, Figure S14: ^1^H NMR spectrum of (6-formylbenzo-1,3-dioxole-5-yl)(2,4,6-trimethylphenyl)iodonium bromide, Figure S15: ^13^C NMR spectrum of (6-formylbenzo-1,3-dioxole-5-yl)(2,4,6-trimethylphenyl)iodonium bromide, Figure S16: ^1^H NMR spectrum of (6-formylbenzo-1,3-dioxole-5-yl)(2,4,6-trimethylphenyl)iodonium tosylate, and Figure S17: ^13^C NMR spectrum of (6-formylbenzo-1,3-dioxole-5-yl)(2,4,6-trimethylphenyl)iodonium tosylate. Block II: RadioTLC chromatograms. Figure S18: RadioTLC analysis of 6-[^18^F]FP obtained via radiofluorination of **4b**, Figure S19: radioTLC analysis of the formulated [^18^F]anle138b.

## Sample Availability

Samples of the compounds **3, 4a, 4b** and [^19^F]anle138b are available from the authors.
