# Supplementary material for: One-Pot Radiosynthesis of [18F]Anle138b—5-(3-Bromophenyl)-3-(6-[18F]fluorobenzo[d][1,3]dioxol-5-yl)-1H-pyrazole—A Potential PET Radiotracer Targeting α-Synuclein Aggregates
Source: Molecules. 2023 Mar 17;28(6):2732. doi: 10.3390/molecules28062732 (PMC10052605; doi:10.3390/molecules28062732)
Supplement: Supplementary file 1 [file molecules-28-02732-s001.zip › molecules-2246232-supplementary.docx]

Supporting information to

**One-pot radiosynthesis of [^18^F]Anle-138b - 5-(3-bromophenyl)-3-(6-[^18^F]fluorobenzo[d][1,3]dioxol-5-yl)-1H-pyrazole -  a potential PET radiotracer targeting α-synuclein aggregates**

V. V. Orlovskaya^1^, O. S. Fedorova^1^, N. B. Viktorov^2^, D.D. Vaulina^1^, R. N. Krasikova^1#^

*^1^ N.P. Bechtereva Institute of Human Brain, 197376 St.-Petersburg, Russia;*

*^2^ St.-Petersburg State Technological Institute (Technical University), 190013, St.-Petersburg, Russia*

1. **NMR and HRMS (ESI) spectra**

**Figure S1**. ^1^H NMR spectrum of 6-fluorobenzo-1,3-dioxole-5-carbaldehyde (400 MHz, CDCl_3_).

**Figure S2**. ^13^C NMR spectrum of **6-fluorobenzo-1,3-dioxole-5-carbaldehyde** (101 MHz, CDCl_3_).

**Figure S3**. ^19^F NMR spectrum of **6-fluorobenzo-1,3-dioxole-5-carbaldehyde** (376.5 MHz, CDCl_3_).

**Figure S4**. ^1^H NMR spectrum of **N'-((6-fluorobenzo-1,3-dioxole-5-yl)methylene)-4-methylbenzenesulfonohydrazide** (400 MHz, DMSO-d6).

**Figure S5**. ^13^C NMR spectrum of **N'-((6-fluorobenzo-1,3-dioxole-5-yl)methylene)-4-methylbenzenesulfonohydrazide (101 MHz, DMSO-d6)**.

**Figure S6**. ^19^F NMR spectrum for compound **N'-((6-fluorobenzo-1,3-dioxole-5-yl)methylene)-4-methylbenzenesulfonohydrazide** (376.5 MHz, DMSO-d6).

**Figure S7**. ^1^H NMR spectrum for compound **[^19^F]Anle138b** (400 MHz, CDCl_3_).

**Figure S8**. ^19^F NMR spectrum of **[^19^F]Anle138b** (376.5 MHz, CDCl_3_).

**Figure S9**. HRMS (ESI) spectrum of **[^19^F]Anle138b**.

**Figure S10**. ^1^H NMR spectrum of compound **4b** (400 MHz, DMSO-d6).

**Figure S11**. ^13^C NMR spectrum of compound **4b** (101 MHz, DMSO-d6).

**Figure S12**. ^1^H NMR spectrum for compound **4a** (400 MHz, DMSO-d6).

**Figure S13**. ^13^C NMR spectrum for compound **4a** (101 MHz, DMSO-d6).

**Figure S14**. ^1^H NMR spectrum of (6-formylbenzo-1,3-dioxole-5-yl)(2,4,6-trimethylphenyl)iodonium bromide (400 MHz, CDCl_3_).

**Figure S15**. ^13^C NMR spectrum of (6-formylbenzo-1,3-dioxole-5-yl)(2,4,6-trimethylphenyl)iodonium bromide (101 MHz, CDCl_3_).

**Figure S16**. ^1^H NMR spectrum of (6-formylbenzo-1,3-dioxole-5-yl)(2,4,6-trimethylphenyl)iodonium tosylate (400 MHz, CDCl_3_).

**Figure S17**. ^13^C NMR spectrum of (6-formylbenzo-1,3-dioxole-5-yl)(2,4,6-trimethylphenyl)iodonium tosylate (101 MHz, CDCl_3_).

1. **RadioTLC chromatograms** (ethyl acetate)

6-[^18^F]FP

[^18^F]fluoride

**Figure S18.** RadioTLC analysis of 6-[^18^F]FP obtained via radiofluorination of **4b.**

[^18^F]anle138b

**Figure S19.** radioTLC analysis of the formulated [^18^F]anle138b.
